# Evaluating the short-term effects of dance on motor and non-motor outcomes in people living with Parkinson’s: A crossover study

**DOI:** 10.1371/journal.pone.0328293

**Published:** 2025-07-31

**Authors:** Anna M. Carapellotti, Matthew Rodger, Michail Doumas

**Affiliations:** School of Psychology, Queen’s University Belfast, Belfast, United Kingdom; Tehran University of Medical Sciences, IRAN, ISLAMIC REPUBLIC OF

## Abstract

**Purpose:**

To investigate the short-term effects of a single dance class on motor and non-motor outcomes in people living with Parkinson’s.

**Methods:**

A crossover design was used. Nineteen people participated. All participants took part in both dance and lecture control conditions seven days apart in a counterbalanced order. Outcomes of gait, balance, functional mobility, and aspects of cognition were measured before and after each condition. A visual analogue scale evaluated self-perceived changes in fatigue, energy, depression, anxiety, concentration, and movement. Statistical analyses were performed using 2x2 repeated-measures ANOVAs with time (pre vs. post) and condition (dance vs. lecture) as factors.

**Results:**

Results showed functional mobility (measured by the Timed Up and Go) improved to a greater extent after dance compared to the lecture. Significant improvements after both dance and lecture sessions were demonstrated for other outcomes measuring balance, cognition, and anxiety, indicating either both conditions had a comparable impact or practice effects may have influenced results.

**Conclusions:**

Further research is needed to confirm the presence and strength of the short-term effect of dance on functional mobility, to assess the impact of dancing on aspects of cognition and anxiety, and to hone the approach to studying the short-term effects of dance.

## Introduction

The benefits of long-term dance practice in people living with Parkinson’s have been extensively studied; however, little is known about short-term effects (i.e., the effects of a single dance class). People living with Parkinson’s who dance have reported short-term benefits in energy, mood, and mobility, and facilitators of these dance classes have also observed such changes [1,2], making it worthwhile to investigate this phenomenon further. Moreover, characterizing the short-term effects of dance may lead to better understandings of the mechanisms underlying the positive results seen over the course of long-term programs.

Thus far, three studies have investigated the short-term effects of dance on motor related outcomes in people living with Parkinson’s. Heiberger et al. [1] showed significant short-term benefits of dance in the MDS-UPDRS motor subscale and found no short-term effects on balance measured using the Semi-tandem test and functional mobility measured using the Timed Up and Go (TUG) test. Bearrs et al. [3] further investigated short-term effects of dance on balance measured using the Berg Balance Scale and functional mobility measured using the TUG, finding significant improvements in balance only. Cameron et al. [2] uniquely measured the short-term effects of dance on movement initiation and bradykinesia using eye tracking, comparing the effect of dancing to the observation of dancing on pro-saccades (automatic eye movements) and anti-saccades (willful eye movements). They found a worsening of pro-saccade performance following dancing and an improvement in anti-saccade performance following the observation of dancing. With small sample sizes, and little overlap between studies, there is limited conclusive evidence for or against short-term motor benefits of dance in Parkinson’s, as measured quantitatively.

A few studies have investigated the short-term impact of dance on other health-related outcomes in people living with Parkinson’s. Prewitt et al. [4] evaluated the short-term effects of dance on several aspects of cognition, demonstrating short-term improvement in executive function, measured using the Fist-Edge-Palm (FEP) test, a sequential manual motor task. Koch et al. [5] measured well-being using the Heidelberg State Inventory, body self-efficacy using the Body Self Efficacy (BSE) scale, and belief in the credibility and expected outcomes of dancing using the Credibility Expectancy Questionnaire, finding all three to improve following one Argentine Tango class. They also measured self-perceived beauty of movements using the BSE beauty subscale and found this to increase, too, suggesting that the aesthetic experience of dance may be an important therapeutic factor. Hadley et al. [6] explored the relationship between body appreciation and well-being in people living with Parkinson’s and the immediate effects of a dance class on these variables. They found that well-being, but not body appreciation, improved following a single class. A qualitative aspect of their study revealed that participation in the dance class seemed to lead to heightened bodily awareness, including awareness of limitations, which may explain why body appreciation did not improve following dancing.

Hulbert et al. [7] also used a mixed methods design to determine if it is possible to ‘see’ (i.e., measure using three-dimensional motion analysis) what is reportedly ‘felt’ (i.e., experienced by people living with Parkinson’s as reported in interviews or conversations) while dancing. Through quantitative measures of biomechanical changes in six movement variables, they found the greatest change from pre- to post-dance in arm movement velocity, degree of trunk rotation, and gait variables in three participants; however, two participants showed changes in similar directions in most outcomes, while one appeared to experience change in the opposite direction (e.g., faster gait speed for two, slower for one). Interestingly, these differences were not reflected in the qualitative data, with all participants reporting favorable effects.

Notably, only one of the aforementioned trials included a control group [2]. The issue of a control group is an important one when measuring quantitatively the short-term effects of dance given the possibility of practice effects or the reliability of the outcome measure influencing results. The relevance of practice effects was highlighted in the study of the short-term effects of aerobic exercise on cognition in people living with Parkinson’s [8,9]. While one uncontrolled study demonstrated positive change in executive function after a single bout of cycling [8], another controlled study found aerobic exercise to have no effect on behavior, suggesting that practice effects may have influenced prior findings [9].

The aim of this study was to characterize the short-term effects of dancing on gait, balance, functional mobility, aspects of cognition, fatigue, energy, depression, anxiety, concentration, and self-perceived ability to move in people living with Parkinson’s, while controlling for social interaction, music, and group learning. The effects of a 60-minute dance class were thus compared to a 60-minute lecture about dance. We hypothesized that improvements in motor and non-motor outcomes would be greater after dancing than after the lecture.

## Materials and methods

This study employed a crossover design. This design was used, as opposed to a randomized controlled trial, because it allowed for a smaller number of participants, which was deemed important by the research team due to knowledge of recruitment challenges associated with this patient population in the region [10]. Moreover, a crossover design meant all participants could serve as their own controls, allowing for greater homogeneity. All participants took part in both the dance and lecture conditions seven days apart in a counterbalanced order. Counterbalancing was achieved by alternating which intervention was delivered first to each group of participants as they signed up in a rolling fashion. Participants were told which condition they would be taking part in prior to attending each session. Before and after each condition (i.e., the dance class or lecture) participants completed a series of tasks measuring gait, balance and cognition, as well as self-report measures. Assessors of these measures were not blinded. Participants on anti-Parkinson’s medication were instructed to take their medication on time on the days they took part in the dance classes and lectures to control for any medication-induced fluctuations.

### Participants

Participants were recruited through Queen’s University Belfast contacts, Parkinson’s UK support groups and referrals, and the community. Inclusion criteria included a diagnosis of Parkinson’s, an ability to walk 10 meters with or without assistance, and an ability to stand for at least 30 minutes. Participants were excluded if they had deep brain stimulation or any major surgeries affecting movement in the past year, any major injuries affecting movement in the last six months, a diagnosis of dementia, or history of serious neurological problems (apart from Parkinson’s). All participants provided written informed consent prior to participating in the trial. The Faculty of Engineering and Physical Sciences Research Ethics Committee of Queen’s University Belfast approved this work (EPS_18_197). Data collection started on April 1^st^ 2019 and ended on June 19^th^ 2019.

### Baseline evaluation

One week prior to participating in the first condition (i.e., the dance class or lecture) participants completed a baseline assessment. Parkinson’s motor symptoms were evaluated using the Movement Disorder Society United Parkinson’s disease Rating Scale III (MDS-UPDRS-III) [11] and the Freezing of Gait Questionnaire (FOG-Q) [12]. A baseline level of cognitive function was evaluated using the Montreal Cognitive Assessment (MoCa) [13,14]. The Activities Specific Balance Confidence Scale (ABC) provided a baseline measure of balance confidence [15]. Symptoms of depression and anxiety were measured using the Hospital Anxiety and Depression Scale (HADS) [16].

### Dance condition

The dance class lasted 60 minutes and followed the three-part format of the Dance for PD® model [17]. It was led by an instructor who had attended the introductory and advanced Dance for PD® training sessions with two years of experience leading such classes herself and an additional year volunteering in a group with an experienced teacher. Detailed descriptions of dance combinations practiced and music used are listed in Table 1.

**Table 1:** Dance combinations practiced in class.

Combination	Music	Description
Seated warm-up		
Gentle warm-up	Frédéric Chopin, Nocturn in C Minor	Port de bras, tendus, slow movements to warm up the torso, arms and legs
Upbeat/rhythmic warm-up	No Music	Warming up legs, arms and torso at a faster tempo; using rhythms/sounds produced with body to synchronize the group
Choreography and storytelling	Leonard Bernstein, Fancy Free: I. Enter Three Sailors	Using choreographic repertoire and imagination to tell a story through movement
Barre exercises		
Plie	Frédéric Chopin, Waltz in E Minor	Plie (knee bends), releve (calf raises) to warm up legs and find balance; port de bras (slow, controlled arm movements)
Tendu	Richard Rodgers, March of the Siamese Children	Reaching and extending through the leg with pointed and flexed feet; practicing weight shifts with legs; coordinating legs and arms
Adagio	Frédéric Chopin, Waltz, Op 70, No 2	Slow controlled leg lifts en croix (front, side, back); testing balance on one leg
Across the floor		
Walking warm-up	Robert Prince, N.Y. Export: Op. Jazz	Walking across the floor to the beat; traveling forwards and on a diagonal; articulating through the feet (ball-heel, heel-toe)
Partner dancing	Leonard Bernstein, Mambo	Learning to dance with a partner and work together; 14 walks forwards and backwards; turning
Circle dance	Jerry Bock, Bottle Dance	Dancing as a group in a circle and supporting each other; stepping sideways (12x), stepping in and out (2x), lifting and lowering arms, repeat L; 8 steps sideways R&L; balancing on one leg to stick one heel into the circle
Improvisational Cool down	Leonard Bernstein, A Day in New York	Participants engaged in mirroring improvisation with a partner, generating their own movements with another dancer following their lead and being the follower themselves

The class began with a 20-minute seated warm-up, followed by 15 minutes of supported balance exercises (i.e., barre exercises), and 20 minutes of dancing across the floor that included traveling in all directions (forwards, backwards, sideways) independently, with a partner, and as a group. The class finished with a five-minute improvisational cool down during which time participants were invited to generate their own movements. All but one combination were performed to music and many incorporated choreographic elements (i.e., learning pre-determined movement sequences). To ensure coherence between information and music presented in the dance class and lecture, a theme was selected, which involved drawing inspiration from the artistic works of a particular choreographer.

### Lecture condition

The control condition was a 60-minute interactive lecture about dance to control for social engagement, learning, and music. The lecture was held at the same time of day as the dance class and was given by the dance instructor. The lecture described the choreographic works and music that served as inspiration for dance combinations practiced in class. It also involved watching YouTube videos of professional dancers performing these works. During the lecture, participants were offered tea or coffee.

Both conditions were attended by one to four volunteers depending on the number of participants in attendance. The volunteers included five university students and an occupational therapist who had recently completed a Dance for Parkinson’s training. Before and after both conditions, participants were given a 15-minute break during which time they were offered water and a snack.

### Outcome measures

**Measures of gait.** The 10 Meter Walk Test (10MWT) measured preferred walking speed. Measurements of gait speed and step frequency taken with the 10MWT are known to be reliable between sessions in people living with Parkinson’s [18]. Participants performed three trials of walking forward at their preferred speed. The time (seconds) and number of steps taken were recorded and averaged, and speed (m/s) was derived. Participants began and stopped walking two meters beyond start and finish lines to control for acceleration and deceleration. During the 10MWT, a Qualisys motion capture system, comprising eight Oqus 3 + cameras running on Qualisys Track Manager software, was used to record movement kinematics of 16 participants by attaching reflective markers to the heel and toe at a sampling rate of 100 Hz. The system was calibrated to achieve a < 1 mm precision of marker position estimation for each capture. The toe marker was placed directly on participants’ shoes and the heel marker was attached to a rigid body that attached to the participants heel and side of the foot in an L shape; a safe adhesive was used to prevent any variability in marker placement between trials.

**Measures of balance and functional mobility.** The 360 degree turn test and TUG measured dynamic balance and functional mobility. For the 360 degree turn test, participants were asked to complete a 360 degree turn to the right (360R) and left (360L); they began by facing a wall and were asked to turn around until they were facing that wall again. This test is known to be a reliable and valid tool for assessing turning ability and fall risk in people living with Parkinson’s [19]. Time to complete the turn and the number of steps taken to turn were recorded [20]. For the TUG [21], participants were asked to stand up from a chair, walk three meters, turn around, and return to sitting in the chair. Time to complete the task was recorded. The TUG has been demonstrated to have high retest reliability [22] and validity [23] in people living with Parkinson’s.

**Measures of cognition.** Trail Making Tests A&B measured visual attention, processing speed, and executive function [24]. Time to complete Trails A&B in seconds and the difference between time to complete Trail B and Trail A were recorded.

The Fist-Edge-Palm test (FEP) involves unimanual sequencing of three hand positions (fist, ulnar edge, palm) and was used to measure motor-sequencing and frontal-executive function [25]. Participants were asked to place on a table top first their fist, then stretched fingers with ulnar side down, and then stretched fingers with palm down in this order as quickly as possible. One point was given for each sequence correctly executed with no mistakes. Points were counted and averaged over two 10-second trials, with preferred and unpreferred hands scored separately. These assessments were videotaped and rescored after each session for accuracy.

The Dual-Task Timed Up and Go (DT-TUG) cognitive was also recorded, with participants completing the TUG while counting backwards by three from a number between 20–100 [26]. DT-TUG is known to enhance the identification of fall risk [27] and is a reliable test in people living with Parkinson’s [22].

The Body Position Spatial Task (BPST) was used to measure the effects of dance on whole-body spatial cognition, motor-cognitive integration, and memory [28]. This outcome is modeled after the Corsi Block Tapping Test, a measure of visuospatial working memory [29]. In the BPST, participants watch an examiner complete movement sequences involving stepping and turning and subsequently attempt to repeat them correctly. Participants were given a practice trial of two moves (“Turn Left, Step Right”). The examiner then began with a trial of two moves, progressing to a maximum of nine moves. There were two trials per level of equal move length, and the number of required moves increased by one at each subsequent level. Participants progressed to the next level if they successfully executed one of the two trials correctly. The span (i.e., the longest movement sequence successfully executed), and the number of levels correctly completed were considered for analysis.

**Self-report measures.** Before and after the dance class and lecture, participants responded to a visual analogue scale (VAS) that evaluated subjective feelings of fatigue, energy, depression, and anxiety, as well as self-perceived ability to concentrate and move on a scale of 1 (indicating ‘not at all fatigued,’ etc.) to 10 (indicating ‘extreme fatigue,’ etc.).

### Data analysis

Statistical analyses were performed using 2 by 2 repeated-measures ANOVAs with time (pre, post) and condition (dance, lecture) as within-subjects factors. In cases where data were not normally distributed the Durbin test was used. Post-hoc comparisons were made using Holm or Conover’s post-hoc tests, respectively. The significance level was set to p < .05. Statistical analyses were performed using JASP (Version 0.14) [30]. Values greater than Q3 + 1.5*Inter-Quartile Range (IQR) or less than Q1-1.5*IQR in a given measure were excluded as outliers from analyses together with the rest of the data points for a given participant in that measure. This process was not applied when the IQR was equal to zero (e.g., BPST) or if the data were non-parametric.

With regard to the motion capture data collected during the 10MWT, position-time trajectories in three dimensions from the right and left toe markers were extracted from Qualisys and imported into MATLAB 2020b for analysis. Kinematic data in x (anterior-posterior), y (medio-lateral), and z (vertical) coordinates were low-pass filtered at 4hz using a Butterworth 5^th^ order dual-pass filter. Trials were then trimmed to ensure that any steps taken outside the 10m path (i.e., the acceleration and deceleration phases) were excluded from the analysis. Data in the vertical dimension were differentiated to calculate velocity, and a fixed threshold for velocity was set to identify the “toe off” point. This point was used to calculate step length and step times. Participant walk ratios (step length[m]/cadence[steps/min]) were calculated from extracted data.

## Results

### Participant characteristics

**Fig 1:**
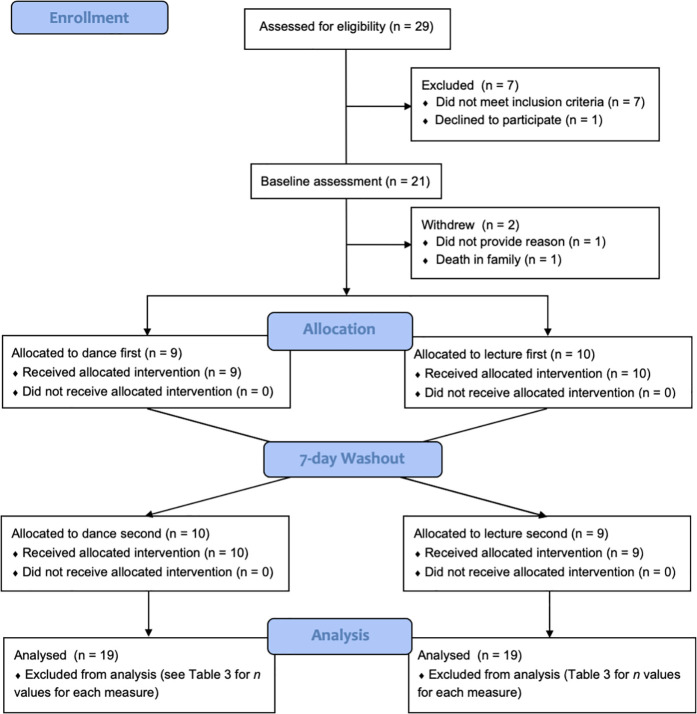
Flow diagram outlining participation.

Thirty-two participants were in contact with the research team regarding this study. Three declined to participate after receiving information, 29 were screened for eligibility, and one further person declined to participate following screening. Twenty-one participants were eligible and agreed to participate. Reasons for exclusion included recent or upcoming major surgeries or injuries, deep brain stimulation, undergoing gradual changes to anti-Parkinson’s medication, or no Parkinson’s diagnosis. Reasons for declining the invitation to participate included participation in another trial, lack of interest, or stress of traveling. Two participants withdrew following the baseline assessment. See figure 1 (i.e., *Participant Flow Diagram*) for the illustrated participation record and table 2 for demographic details of the 19 participants who completed the study.

**Table 2:** Demographic characteristics of participants.

Demographic characteristics (n = 19)	Values	
	Mean	SD
Age (years)	67.84	6.24
Education (years)	15.05	3.94
MoCa	26.42	2.09
Duration of disease (years)	7.38	7.16
Modified Hoehn & Yahr	2.03	0.90
MDS-UPDRS III	33.11	14.79
ABC Scale	70.79	18.29
HADS (total score)	10.47	4.13
FOG-Q	8.42	5.44
	*n*	*n*
Gender (male/female)	12	7
Therapeutic dance experience? (Y/N)	9	10
Formal dance training? (Y/N)	3	16
Falls in the past year? (Y/N)	5	14
Anti-parkinson medication? (Y/N)	17	2

*Notes.* MoCA, Montreal Cognitive Assessment, MDS-UPDRS III, Movement Disorder Society United Parkinson’s Disease Rating Scale III, ABC, Activities-Specific Balance Confidence Scale, HADS, Hospital Anxiety and Depression Scale, FOG-Q, Freezing of Gait Questionnaire.

The time since last L-dopa dose was recorded and any differences analyzed with a 2 by 2 ANOVA. There was no main effect of condition on medication state nor was there a condition by time interaction. There was a main effect of time on medication state, with less time between the last dose and the post-test assessment due to the fact that many participants took medication at some point during the experiment; F(1,15) = 6.377, p = .023, partial η^2^ = .29.

Nine participants completed the dance condition first and 10 participants completed the lecture condition first. The lecture or dance classes were delivered in a minimum group of one participant and a maximum group of four. One participant was unable to complete assessments of mobility and motor-cognitive outcomes, including 10MWT, 360 degree turn test, TUG, DT-TUG, and BPST due to severe freezing episodes, despite being able to complete the dance class, and another was not able to complete the BPST and DT-TUG because the assessment session had to be terminated when a research assistant became suddenly ill.

### Outcome measures

Table 3 lists the statistical tests used for each measure, *n* values (reflecting the number of outliers removed and missing data points), means and SDs of all outcomes (pre and post dance and lecture conditions), and *p* values for simple main effects and interaction effects.

**Table 3:** Table of all pre and post values, p values and effect sizes for simple main effects and interaction effects.

Outcome	Pre Lecture	Post Lecture	Pre Dance	Post Dance	p-value condition	p-value time	p-value interaction
***Clinical measures (n =)***							
10MWT (sec) (17)*	4.69 (0.61)	4.64 (0.62)	4.67 (0.63)	4.58 (0.51)	0.54 [0.02]	0.54 [0.02]	N/A
10MWT (steps) (17)*	10.51 (1.41)	10.57 (1.09)	10.53 (1.18)	10.29 (0.96)	0.68 [0.01]	0.31[0.06]	N/A
TUG (sec) (15)	9.95 (1.47)	9.74 (1.29)	10.23 (1.37)	9.42 (1.50)	0.92 [<0.01]	0.002 [0.51]	**0.049 [0.25]**
360R (sec) (16)	3.86 (1.21)	3.77 (1.15)	3.83 (1.17)	3.64 (1.13)	0.52 [0.03]	0.24 [0.09]	0.52 [0.03]
360R (steps) (17)	8.56 (2.09)	8.41 (1.49)	8.24 (1.70)	8.06 (1.85)	0.18 [0.11)	0.51 [0.03]	0.95 [<0.01]
360L (sec) (18)	4.16 (1.82)	3.94 (1.45)	4.11 (1.22)	3.80 (1.23)	0.63 [<0.01]	**0.046 [0.21]**	0.74 [<0.01]
360L (steps) (17)	8.88 (3.00)	8.59 (1.91)	8.77 (2.10)	8.03 (1.61)	0.47 [0.03]	0.140 [0.13]	0.52 [0.03]
***MoCap measures (n =)***							
Gait Speed (m/sec) (16)	1.21 (0.14)	1.21 (0.15)	1.20 (0.13)	1.20 (0.12)	0.68 [<0.01]	0.96 [<0.01]	0.85 [<0.01]
Cadence (15)	111.13 (6.99)	112.15 (6.25)	111.39 (8.24)	110.46 (7.17)	0.45 [0.04]	0.97 [<0.01]	0.28 [0.08]
Step Length (cm) (16)	64.30 (7.80)	63.99 (7.94)	64.15 (6.93)	64.63 (5.62)	0.78 [<0.01]	0.89 [<0.01]	0.45 [0.04]
Walk Ratio (cm/steps/min) (16)	0.58 (0.09)	0.57 (0.09)	0.58 (0.09)	0.58 (0.09)	0.39 [0.05]	0.59 [0.02]	0.26 [0.08]
***Cognitive measures (n =)***							
Trail A (sec) (17)	32.98 (11.30)	29.28 (11.13)	34.80 (14.17)	31.28 (11.58)	0.38 [0.05]	**0.018** [0.30]	0.93 [<0.01]
Trail B (sec) (15)	88.52 (41.01)	67.58 (23.36)	72.46 (27.14)	66.45 (24.45)	0.23 [0.10]	**0.007** [0.41]	0.10 [0.19]
Trail B-A (sec) (15)	57.21 (34.15)	40.33 (15.76)	41.36 (22.16)	37.74 (19.56)	0.16 [0.13]	**0.028** [0.30]	0.136 [0.15]
FEP (preferred) (18)*	6.75 (1.97)	7.03 (1.91)	6.47 (1.32)	6.86 (1.66)	0.63 [0.02]	0.34 [0.05]	N/A
FEP (unpreferred) (18)*	6.61 (1.57)	7.06 (1.90)	6.64 (1.53)	7.06 (1.57)	0.81 [<0.01]	**0.032** [0.33]	N/A
BPST (level) (16)	2.56 (0.73)	2.75 (0.68)	2.50 (0.73)	3.25 (0.86)	0.11 [0.16]	**0.016** [0.33]	0.108 [0.16]
BPST (span) (16)	3.56 (0.73)	3.75 (0.68)	3.50 (0.73)	4.25 (0.86)	0.11 [0.16]	**0.016** [0.33]	0.108 [0.33]
DT-TUG (sec) (15)	12.51 (2.28)	12.04 (2.56)	12.30 (2.41)	11.79 (2.40)	0.54 [0.03]	0.071 [0.21]	0.93 [<0.01]
***VAS scale (n =)***							
Fatigue (14)*	3.86 (2.51)	3.64 (2.50)	3.71 (2.46)	3.64 (2.50)	1.00 [<0.01]	1.00 [<0.01]	N/A
Energy (14)*	6.50 (1.99)	6.42 (2.31)	6.00 (2.25)	6.79 (2.39)	0.46 [0.04]	0.31 [0.07]	N/A
Depression (14)*	2.07 (1.73)	1.64 (1.08)	1.71 (1.14)	1.57 (0.94)	0.71 [0.01]	0.39 [0.05]	N/A
Anxiety (14)*	2.86 (1.75)	1.93 (1.14)	2.36 (1.28)	1.93 (1.14)	0.76 [0.07]	**0.049** [0.28]	N/A
Movement (14)*	4.21 (1.85)	3.43 (1.79)	4.29 (1.98)	3.50 (1.99)	0.65 [0.07]	0.084 [0.21]	N/A
Concentration (14)*	3.50 (1.87)	4.00 (2.25)	4.93 (1.86)	4.29 (2.67)	0.12 [0.17]	0.52 [0.03]	N/A

*Note*. Pre- and post-values are means and standard deviations (in brackets). Asterisks (*) indicate outcomes analyzed using the non-parametric Durbin test. All other outcomes were analyzed using 2 by 2 Repeated Measures ANOVAs. Values in square brackets next to p-values are estimates of effect size (partial η^2^ for ANOVAs and ε^2^ for the Durbin test). The *n* values reflect the number of participants included in each analysis.

**Measures of gait.** For the 10MTW test, including time to complete (sec) and number of steps, there were no differences between condition or time factors. For motion capture measures of gait, there were no main effects of condition or time nor interaction effects for gait speed (m/s), cadence, step distance (cm), or the walk ratio.

**Measures of balance and functional mobility.** For the 360 turn test, including time to complete (sec) and number of steps, there were no main effects of condition in either direction (R or L). There was a main effect of time on 360L time (sec), reflecting faster completion of the test after both conditions (*M* change = −0.263 secs, averaged over levels of condition); F(1,17) = 4.618, p = .046. There were no other main effects of time and no interaction effects.

**Fig 2:**
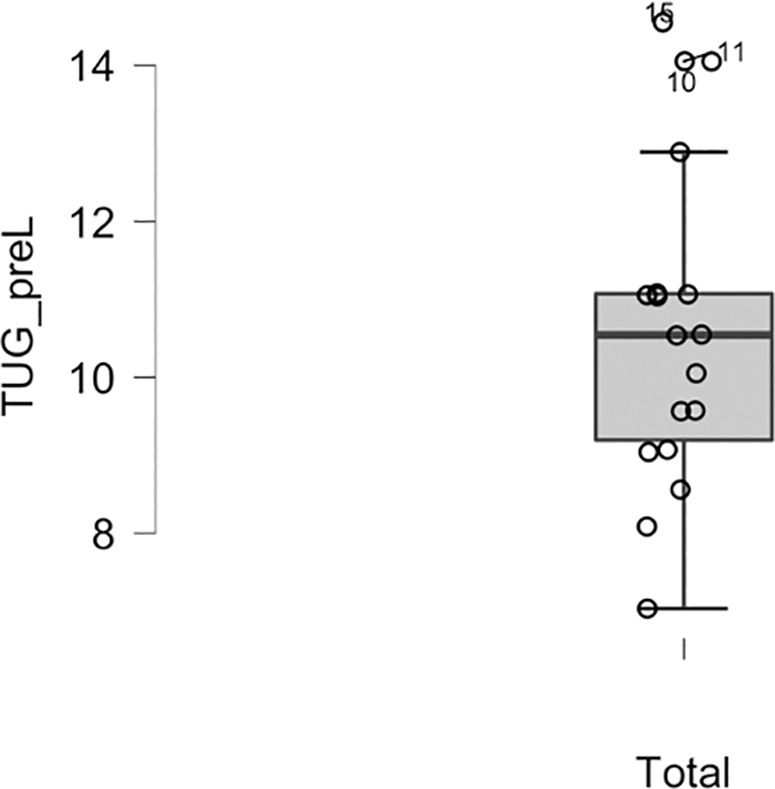
Outliers removed for TUG. Outliers (points 10, 11, 15) are values greater than Q3 + 1.5*Inter-Quartile Range (IQR) or less than Q1-1.5*IQR.

**Fig 3:**
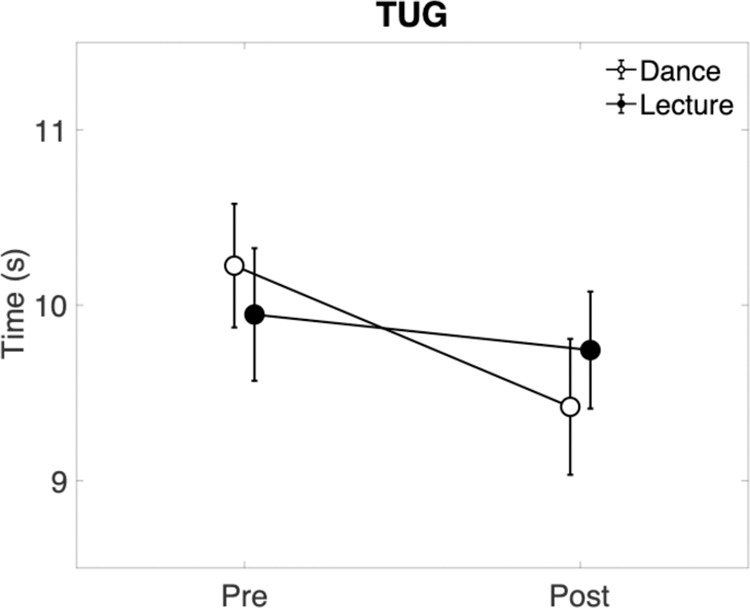
Scores for TUG. Values are means and error bars reflect ± 1 standard error of the mean.

For the TUG test (see Figs 2 and 3), a 2 by 2 repeated-measures ANOVA revealed a significant Time by Condition interaction, *F*(1, 14) = 4.64, p = .049. Post hoc comparisons with Holm-Bonferroni correction showed that TUG performance improved significantly from pre to post in the dance condition (p = .002, *M* change = −0.81 s), but not in the lecture condition (p > .05, *M* change = −0.20 s). Additionally, there was a significant main effect of Time, *F*(1, 17) = 14.54, p = .002, indicating overall faster TUG performance at post-test (*M* change = −0.50 s, averaged across conditions). While the main effect of time indicated an overall improvement, post hoc tests confirmed that this was driven exclusively by the dance condition. The main effect of Condition was not significant (p > .05).

**Measures of cognition.** There were no main effects or interaction effects for the DT-TUG. There were no main effects of condition nor interaction effects for Trail A (sec), Trail B (sec), Trail B-A, BPST level and span, or FEP (preferred or unpreferred).

For the Trail Making Test, a decrease in score indicates an improvement on the task. There was a main effect of time on Trail A; F(1,16) = 6.880, p = .018, with time to complete the task (sec) decreasing after lecture and dance (*M* change = −3.607 secs, averaged over levels of condition). Similarly, there was a main effect of time for Trail B; F(1,14) = 9.746, p = .007, with mean scores decreasing after lecture and dance (M change = −13.471 secs, averaged over levels of condition). There was a main effect of time on Trail B-A; F(1,14) = 6.016, p = .028, with the mean difference in scores decreasing after lecture and dance (M change = −10.249 secs, averaged over levels of condition).

For the BPST, an increase in score indicates improvement on the task. There was a main effect of time on the BPST level; F(1,15) = 7.289, p = .016, with mean scores increasing over time (M change = 0.469, averaged over levels of condition). There was also a main effect of time on BPST span; F(1,15) = 7.289, p = .016, with mean scores increasing over time (M change = 0.469, averaged over levels of condition).

For FEP (preferred and unpreferred), an increase in score indicates improvement on the task. There was an effect of time for the unpreferred side only, χ^2^(1, n = 18) = 4.614, p = .032, with a slight increase in scores for lecture and dance (*M* change = 0.431, averaged over levels of condition).

**Self-report measures.** There were missing data points for these outcomes due to researcher errors. Pre-lecture there were four missing data points and pre-dance there was one missing data point for all questions of the VAS. Pre-dance and post-dance there was one missing data point for the final three questions. Only complete datasets were included in the analysis.

There were no effects due to condition or time factors for VAS measures of fatigue, energy, depression, concentration or movement effort. There was a significant effect of time for anxiety, χ^2^(1, n = 14) = 3.882, p = .049, which decreased after lecture and dance (*M* change = −0.679, averaged over levels of condition).

## Discussion

The aim of this preliminary study was to assess the effects of a single dance class on motor and non-motor outcomes in people living with Parkinson’s. Our results showed that time to complete the TUG task improved to a greater degree after dancing compared to attending the lecture session, and significant improvements after both dance and lecture sessions were demonstrated for other motor and non-motor outcomes, including the 360 turn test (L, secs), Trail A, Trail B, Trail B-A, BPST (span and level), FEP (unpreferred), and anxiety.

Our main finding shows that the time to complete the TUG task decreased to a greater extent after the dance class (M change = −0.81s) in comparison to the lecture (M change = −0.20s). This decrease in time following dancing is not in agreement with two previous studies, which showed no significant reduction in time to complete this task [1,3]. This finding in our study should be interpreted with caution for several reasons. First, the improvement seen in the TUG was less than one second (M change = −0.81s), with a *p* value just below 0.05. The minimal important difference of the TUG in people living with Parkinson’s has been described as 3.5 seconds [31]; however, it is unknown what level of change would be realistic or meaningful following an effective one-hour intervention. Furthermore, placebo response rates are known to be high in people living with Parkinson’s due to an expectation of improvement being linked to a release of dopamine that could in turn potentially result in the clinical improvement of motor symptoms [32]. It is also important to note that this interaction was shown in our study following the removal of three outliers, who were three of the only participants living with H&Y stage III Parkinson’s. This perhaps implies that such short-term gains in mobility following physical activity may become more difficult as Parkinson’s progresses. Only one other measure of mobility, time to complete the 360 degree turn test (L), showed a main effect of time, that is, the average time-to-complete this test was quicker after the dance and lecture conditions.

This study also showed a main effect of time (from before to after each condition) for several cognitive outcomes, including Trail Making Tests, BPST and FEP. For the FEP (unpreferred hand), the number of correctly executed sequences increased from pre- to post-test in both conditions. Prewitt et al. [4] previously found the FEP to improve after one hour of dancing in their uncontrolled study. The fact that both conditions led to improvement in our study calls into question whether the effects of the dance class and the lecture were comparable or if practice effects influenced this particular outcome here and in prior research. Trail Making Tests and BPST similarly showed improvement after both the dance class and lecture, again suggesting that practice effects may have influenced results. The presence of similar short-term effects of time in the dance and lecture conditions in cognitive outcomes is novel and important in that it suggests that a single dance class, at least in the way it was delivered in our study, is not sufficient to produce beneficial effects over and above the effects of a lecture control condition.

Cameron et al.’s [2] study showed a worsening of pro-saccade performance (automatic eye movements) following dancing and an improvement in anti-saccade performance (willful eye movements) following the observation of dancing. They suggest that the observation of dancing resulted in plasticity changes in brain areas important for executive control, while dancing benefited voluntary mobility at the expense of automatic movements. Given that our control condition included watching videos of dancing, it is plausible that the cognitive measures, all of which involved movement (i.e., drawing, executing movement sequences), improved in both conditions through two different mechanisms: via engagement of the mirror neuron system after the lecture and via voluntary movement signals benefiting mobility after dancing [2]. Further research, such as a comparative study with an inactive control, will thus be necessary to confirm the existence of short-term effects of dancing or observing dance on executive function, spatial cognition, and motor-cognitive integration. An active control, such as an exercise intervention practiced to music, may also be useful in confirming any effects, as it remains a challenge to find a well-matched control group for an activity as multidimensional as dancing.

Our study compared means across time points and conditions for all outcomes, which may mean that some individual benefits were left unseen. Hulbert et al. [7] took an individualized approach by analyzing the short-term biomechanical changes of six movement variables of three dance class participants living with Parkinson’s. They found two participants showed changes in similar directions in most outcomes (e.g., gait variables), while one appeared to experience change in the opposite direction (e.g., faster gait speed for two participants, slower for one). These differences were not reflected in the qualitative data, with all participants reporting positive effects of dancing. The authors conclude that it may not be the improvement in physical ability (as defined by the researchers) that is important, but unique perceptions of change or the experience of new movement qualities that are meaningful [7]. We did not ask participants about their experiences of the dance classes, the testing sessions, or their effects so we cannot confirm if the quantitative results match their perceptions. Participant perceptions and experiences would also have been useful for other findings in our study, such as the decrease in anxiety. Qualitative approaches or multidimensional measurement tools may have also been valid and more informative ways to assess any changes in non-motor aspects of Parkinson’s disease, as opposed to a VAS, which was quick and feasible to administer in this context but limited in terms of the amount of information it can provide.

### Limitations

One potential limitation of this study is that the study design necessitated that the dance classes take place in a laboratory on campus and in small groups of one to four participants. It was a challenge creating an atmosphere equivalent to community-based dance classes in this context. One participant, a regular in community-based dance classes at the time, commented after the class that it “felt different.” Future studies investigating short-term effects should therefore gather feedback from seasoned dancers during the study design stage to ensure that the experience is comparable to that of a typical dance class environment.

Other potential limitations of this study may include a lack of statistical power due to the small sample size or the large number of outcomes, which may have diluted some findings. Participants in previous research studies have reported improved mood, mobility and energy following dancing [1,7]. Though neither fatigue nor energy were impacted by either condition, completing 15 minutes of physical and mental tests before and after the dance class may have washed out any potential energizing effects of dance. Relatedly, although a number of movement-based assessments were carried out with participants, we cannot be certain that these would have been sensitive to the relevant characteristics of action that may be modulated through dancing. Kinematic qualities of movement coordination across limbs, or action-selection preferences, would be unlikely to show up in our measures, yet may be meaningfully modulated by dancing.

Furthermore, it remains difficult to ascertain how meaningful any short-term changes in the outcome measures actually are. Future studies should consider taking baseline measurements of outcomes of interest to further confirm each measurement tool’s validity and reliability beyond its known psychometric properties, especially for aspects of cognition as there is currently little evidence demonstrating the reliability of cognitive measures frequently used in studies of Parkinson’s. Due to a lack of evidence, it was not possible to be certain of the amount of time needed between the two conditions for the effects of either intervention on the outcomes to be completely “washed-out,” therefore, a randomized controlled trial with a parallel design could be used to strengthen conclusions. Randomization regardless of study design, blinding the outcome assessors, and an intention-to-treat analysis will also be important in ensuring validity and reliability in future studies, as the lack of such controls may have introduced biases here. Participants expectations of the interventions should also be assessed in future trials to evaluate the influence of any placebo effects.

## Conclusions

This controlled investigation of the short-term effects of dance on motor and non-motor outcomes in people living with Parkinson’s demonstrated improvement of functional mobility following dancing and showed improvements over time for other outcomes. Further research will be needed to confirm the presence and strength of the short-term benefits of dance on mobility, to assess the short-term effects of dancing and observing dance on aspects of cognition, and to hone the approach to measuring short-term effects of dance. The findings of our study and new understandings in this research area highlight the methodological challenges of quantitatively capturing the short-term benefits of dancing in people living with Parkinson’s. Future experimental paradigms will need to consider the features of dance classes that potentially underly commonly reported benefits and ensure that these remain present in the context of a laboratory-based investigation. As demonstrated by Hadley et al. [6] and Hulbert et al. [7], the value of experiential insights in supporting our understanding of measured outcomes in this context is clear. Investigating dance in natural rather than laboratory-based settings may also be a valuable way of capturing the short-term effects of dance, as well as the ephemeral experience of dancing that leads to them.

## Supporting information

S1 File Analyzed data. Spreadsheet with data included in statistical analyses.
(XLSX)
